# Variable importance for sustaining macrophyte presence via random forests: data imputation and model settings

**DOI:** 10.1038/s41598-018-32966-2

**Published:** 2018-09-28

**Authors:** Wout Van Echelpoel, Peter L. M. Goethals

**Affiliations:** Department of Animal Sciences and Aquatic Ecology, Ghent University, Coupure Links 653, B-9000 Ghent, Belgium

## Abstract

Data sets plagued with missing data and performance-affecting model parameters represent recurrent issues within the field of data mining. Via random forests, the influence of data reduction, outlier and correlated variable removal and missing data imputation technique on the performance of habitat suitability models for three macrophytes (*Lemna minor*, *Spirodela polyrhiza* and *Nuphar lutea*) was assessed. Higher performances (Cohen’s kappa values around 0.2–0.3) were obtained for a high degree of data reduction, without outlier or correlated variable removal and with imputation of the median value. Moreover, the influence of model parameter settings on the performance of random forest trained on this data set was investigated along a range of individual trees (*ntree*), while the number of variables to be considered (*mtry*), was fixed at two. Altering the number of individual trees did not have a uniform effect on model performance, but clearly changed the required computation time. Combining both criteria provided an *ntree* value of 100, with the overall effect of *ntree* on performance being relatively limited. Temperature, pH and conductivity remained as variables and showed to affect the likelihood of *L*. *minor*, *S*. *polyrhiza* and *N*. *lutea* being present. Generally, high likelihood values were obtained when temperature is high (>20 °C), conductivity is intermediately low (50–200 mS m^−1^) or pH is intermediate (6.9–8), thereby also highlighting that a multivariate management approach for supporting macrophyte presence remains recommended. Yet, as our conclusions are only based on a single freshwater data set, they should be further tested for other data sets.

## Introduction

Aquatic macrophytes are an essential component of freshwater communities as their role in providing food and shelter has long been recognised^1–4^. Moreover, their presence has been linked to ecological benefits occurring at local, including sediment stabilisation, protection against waves and oxygenation and at regional scale, including the slowing down of hydrological surges and improving the efficiency of biochemical cycles^1,5^. These benefits combine into specific ecosystem functions and accompanying services, for instance clear water and supporting biodiversity^6–8^, claimed by humanity to provide in its daily needs. Preservation of these key functions has been a main area of focus lately, requiring a sound knowledge of (i) variables that influence macrophyte presence, (ii) type of water body considered, and (iii) functional traits of macrophytes^2,9,10^. In short, macrophyte presence is in the first place affected by habitat suitability, which determines whether natural establishment or manual introduction will be successful and lead to a self-sustaining community.

Suitable habitats are species-specific and characterised by environmental conditions reflecting the optimal, preferred conditions, which are represented by a habitat suitability index (HSI) close to unity. In contrast, a HSI value close to zero represents a highly suboptimal habitat for one species, while potentially being (close to) optimal conditions for another species. Determination of optimal conditions and related habitat suitability indices is frequently performed with habitat suitability models (HSM), which allow to fill in the gaps in current ecological knowledge about variable importance and provide predictions for future species distributions^11,12^. For instance, Kemp, *et al*.^13^ analysed the habitat requirements of submerged aquatic vegetation in the Chesapeake Bay and found that light availability is the most influential variable, while being influenced by nutrient concentrations and suspended sediments.

Different types of HSM exist, positioned along an axis between data-driven (empirical) and knowledge-driven (conceptual) models^14^, without a single best modelling approach, as a universal grading of HSMs has not yet been proven successful^15,16^. So far, despite their limited ecological relevance and vulnerability to data imperfections and uncertainties^17,18^, data-driven models have been applied and compared frequently when forecasting habitat suitability and species distributions^19–21^. Moreover, novel data-driven modelling techniques are continuously developed or adopted from other disciplines, with improved accuracies and limited drawbacks. For instance, random forests were widely used in bioinformatics before being applied in ecology and provide convincing results related to classification accuracy, the ability to model interactions and to determine variable importance^22^. Random forests is a machine learning technique and belongs to the group of decision trees (DTs), of which classification and regression trees (CARTs) are well known examples, considering their frequent application in ecological studies^23,24^. Drawbacks of the application of CARTs include the creation of complex trees when large data sets are used, the limited ability of including ecological knowledge and the potential of overfitting^17^. Moreover, data preprocessing and model parameterisation influence model performance, hence requiring bagging or the application of a range of settings to allow an objective comparison of the developed models^25,26^. For more information on CART, we refer to Rokach^27^ and Van Echelpoel, *et al*.^17^ and references therein.

Random forests include the required bagging process and have been successfully applied for inferring habitat suitability and distribution of fish, plants and macroinvertebrates^19,28,29^. Yet, both data preprocessing and parameter settings still influence final model performance and have to be considered throughout. This has been illustrated by Everaert, *et al*.^25^ who considered the use of a data set either with or without subsampling for decision tree training. The first set had a skewed distribution among the response classes and was randomly subsampled to create a uniformly distributed second data set. Subsequently, they changed the number of cross-validations, the complexity parameter and the required minimum number of objects within the leaf of the decision tree prior to further splitting. Based on these combinations, they observed that a uniform distribution of the response classes is beneficial for overall model performance, while a higher number of cross-validation folds resulted in more unstable performances. Hence, a preliminary analysis of the model parameters’ effect on model performance is recommended, especially because the majority of data sets is plagued by a certain degree of missing data. Some researchers opt for a complete removal of the observations with missing data, although in some cases this can result in an unwanted amount of data being removed or the introduction of bias when investigating associations. To overcome this, a plethora of imputation techniques have been suggested during the past decades, starting with univariate imputation methods like mean and median imputation up to more recent, multivariate methods like *k*-nearest neighbours (kNN), multiple imputed chained equations (mice), Bayesian principal component analysis (BPCA) and iterative random forest-based imputation (e.g. *missForest*)^30–32^. Similar to HSMs, no single best imputation technique has yet been identified, as this is likely to depend on the type of data being considered.

The aim of this experiment is to focus on the influence of data preprocessing and model parameter selection on the identification of suitable habitats for macrophytes by using habitat suitability models. To do so, we will (i) consider different combinations of data preprocessing steps, including data reduction, outlier and correlated variable removal and imputation method, (ii) determine the effect of model parameters on overall model performance and (iii) analyse the effect of a single variable on the likelihood of macrophyte presence. Robustness and stability will be determined via repetitive model development and evaluation (10 times). This work contributes to the existing knowledge related to (i) the effect of data preprocessing and model parameterisation on model performance and (ii) the influence of abiotic water variables on the likelihood of macrophyte presence.

## Results

### Obtained dataset

**Figure 1:**
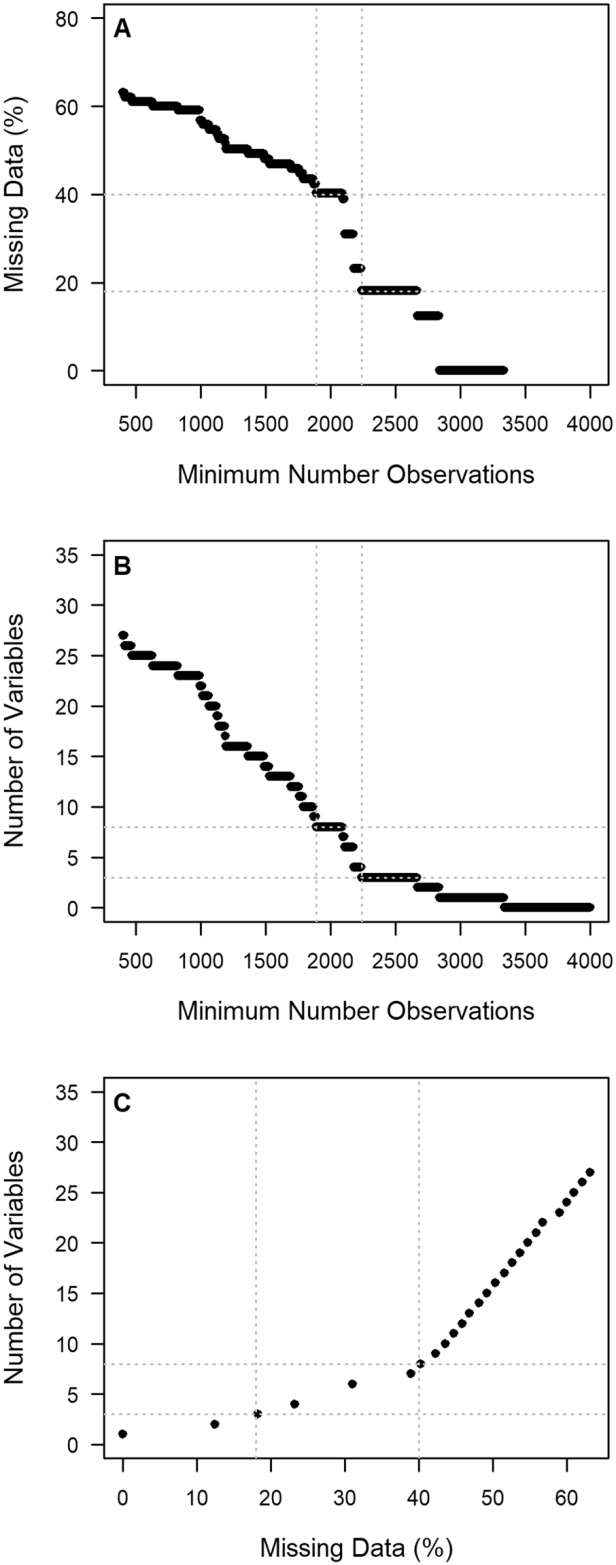
Effect of threshold selection on the relative proportion of missing data and the number of remaining variables. With increasing threshold levels (‘Minimum Number of Observations’), more limitedly represented variables are removed, thereby decreasing the amount of missing data (**A**) and remaining variables (**B**). A high number of variables has to be removed to obtain a high decrease in missing data (**C**). The dashed lines represent the additional data sets to be used for evaluating model performance and the effect of model settings. Thresholds were based on visual inspection and obtained drop in missing data.

In total 4344 unique space-time combinations were found to be present within the Limnodata database. Data of 174 chemical variables were included, yet were not observed for each sample, amounting to 94% of missing data points. Reduction based on a minimum number of observations showed that, by increasing this threshold, a gradual decrease in both missing data and number of variables was observed (see Fig. 1A,B). For instance, starting with a threshold of 430 values (i.e. 10% of the total number), the number of variables was reduced to 26, while the proportion of missing data had reduced to 62%. Gradually reducing the number of variables to 8 also gradually reduced the amount of missing data to 40%, followed by even bigger missing data reductions by removing additional variables (Fig. 1C). Final data set selection was based on minimal, intermediate and extensive removal of both variables and missing data, and were chosen arbitrarily based on Fig. 1. We defined our starting point (i.e. a variable should have at least a reported value for 10% of the overall number of instances) as the data set with minimal removal of variables. Secondly, we defined the elbow location (Fig. 1C), occurring around 8 variables and 40% missing data as the data set with intermediate removal of variables (missing data drops from 62% to 40%). Lastly, we defined the number of variables resulting in a similar drop of missing data (i.e. from 40% to around 18%) to be the data set with extensive removal of variables. The three selected data sets contained 4327 observations of 26 variables (62% missing), 4107 observations of 8 variables (40% missing) and 3604 observations of 3 variables (18% missing) and will further on be referred to as M62, M40 and M18, respectively.

Data preprocessing resulted in further data reduction, yet no strong correlations were found among the three remaining variables of the M18 data set, hence creating similar data sets as the ones without correlation analysis. After data imputation, 40 unique data sets were obtained and linked to presence/absence information of three macrophytes with an original prevalence of 26% (*Lemna minor*), 18% (*Spirodela polyrhiza*) and 10% (*Nuphar lutea*).

### Model performance

**Figure 2:**
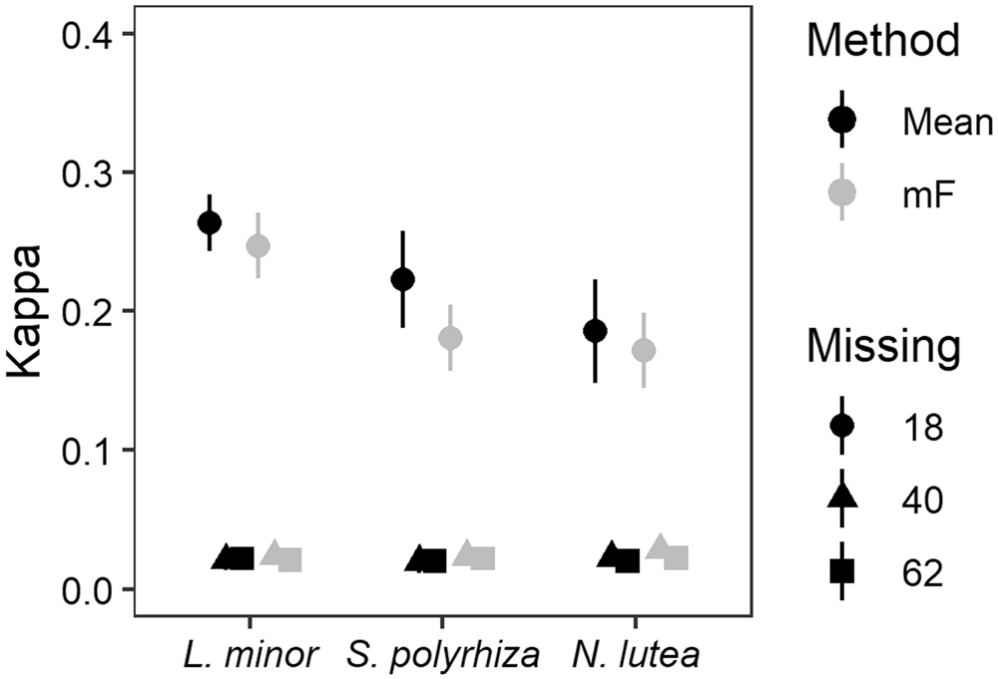
Effect of missing data on random forest performance. Higher performances are observed when the original amount of missing data is low and prevalence is relatively high. In contrast, no clear effect of imputation method on random forest performance can be observed (shown methods including the univariate Mean imputation and multivariate missForest (mF) based imputation). Depicted performances were obtained with random forest consisting of 100 trees, while running 10 repetitions and applying a 5-fold cross-validation. Selected data sets underwent outlier and correlated variable removal prior to model development.

Cohen’s kappa was highly influenced by the degree of missing data within the original data set as clearly higher values (up to 0.30) were observed for M18, while its value was close to 0 in case of M40 or M62 (see Fig. 2). Within the former case, higher performances were observed for each of the three macrophytes, along with an additional negative influence of decreasing prevalence. Moreover, no clear difference between univariate and multivariate imputation method on model performance was suggested to be present. These observations are illustrated in Fig. 2, showing random forest performance for *L*. *minor*, *S*. *polyrhiza* and *N*. *lutea* with three different degrees of missing data (settings: *ntree* = 100, 5-fold CV, 10 repetitions). To ease visual interpretation, only a subset of the results was selected. More specifically, outlier and correlated variable removal were performed and imputation of a univariate imputation (mean) or via a multivariate approach (*missForest*) occurred. Additional supporting graphs can be found in Supplementary Information (Figs S1–S3). Subsequent result analysis will be based on the M18 data set.

**Figure 3:**
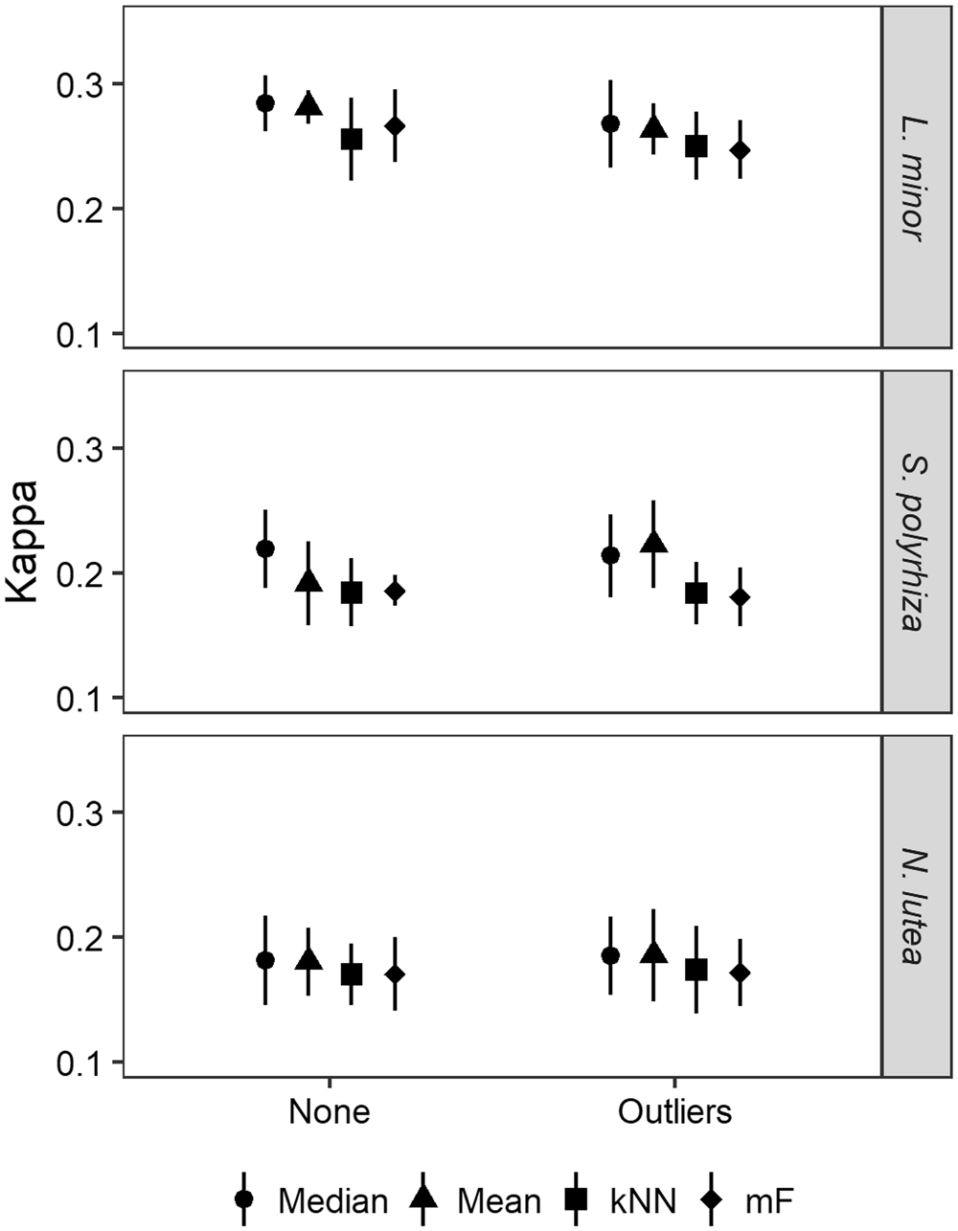
Effect of data preprocessing and imputation method on random forest performance. Similar performances are obtained for each combination, with multivariate imputation methods (k nearest neighbours (kNN) and missForest (mF)) performing slightly worse than univariate imputation methods (mean and median value). Depicted performances were obtained with random forest consisting of 100 trees, while running 10 repetitions and applying a 5-fold cross-validation. Selected data sets originally consisted of 18% missing data and underwent either no (‘None’) preprocessing or outlier removal (‘Outliers’) prior to model development.

Without any strong correlation among the three variables, only eight unique data sets remained, representing the four imputation techniques applied on the data without preprocessing or with only outlier removal. No clear positive effect of any of the imputation techniques nor data preprocessing on model performance could be inferred, as all combinations provided Kappa-values between 0.18 and 0.30 (settings: *ntree* = 100, 5-fold CV, 10 repetitions), being illustrated in Fig. 3. In general, multivariate imputation methods (kNN and *missForest*) seemed to perform slightly worse than univariate imputation methods (mean and median).

These results allowed to select the following data preparation procedure for this specific case: (1) reduction of the data down to 18% missing data points, with (2) no outlier and correlated variable removal and followed by (3) replacing missing data via a univariate imputation approach (e.g. the median). Based on this data set, the following sections deal with (1) the effect of model parameter settings (*ntree* and *mtry*) on model performance, (2) the error between the observation and predicted likelihood and (3) the influence of an environmental variable on the likelihood of a macrophyte being present.

### Parameter settings

**Figure 4:**
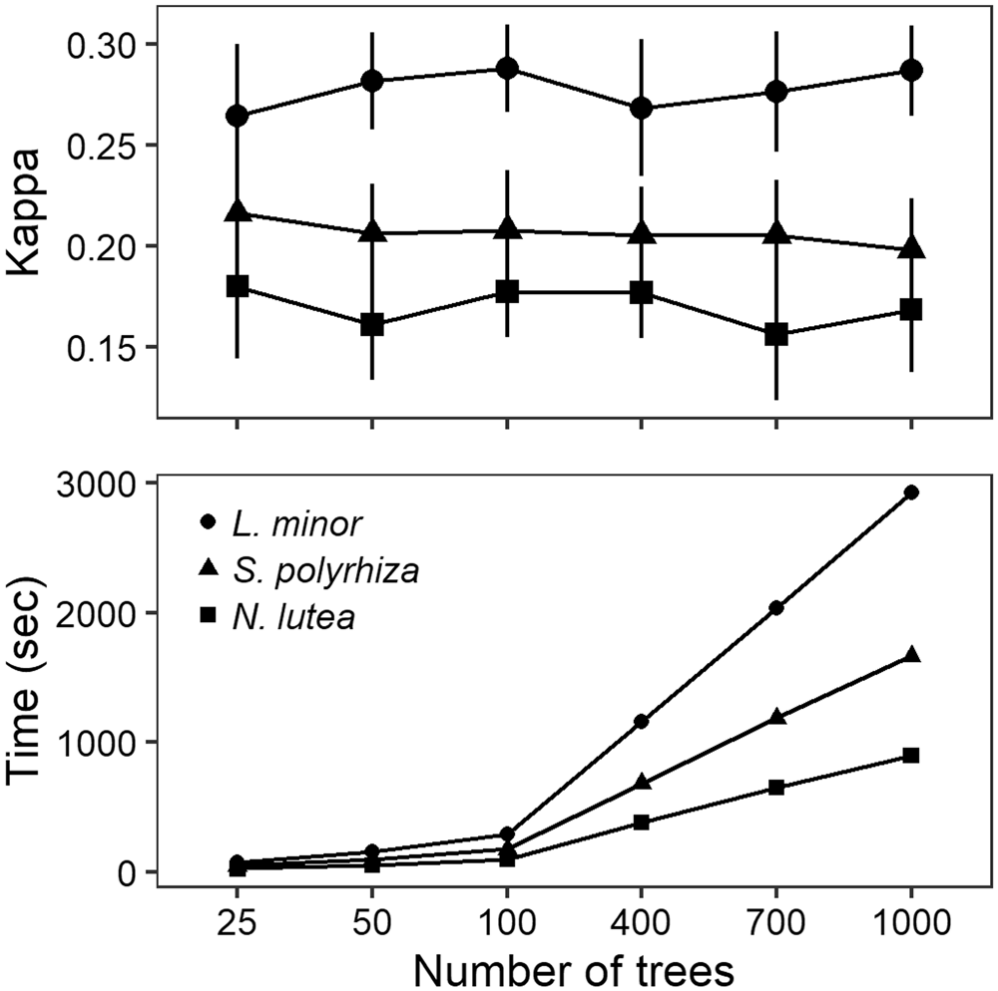
Effect of individual trees (ntree) on model performance and computation time for Lemna minor, Spirodela polyrhiza and Nuphar lutea. Along an increase in number of individual trees, no clear increase in kappa values can be observed, while a clear increase in computation time is present. Performance values were calculated for a random forest trained with data of which 18% was missing, without outlier and correlated variable removal, followed by median imputation. During model training, 5-fold cross-validation was applied and repeated with ten different runs, for which the average computation time was determined.

Altering the number of individual trees to be developed in the random forest, did not uniformly change the performance of the model (settings: 5-fold CV, 10 repetitions). For instance, a random forest developed for *L*. *minor* had an average default performance of kappa = 0.29 (+/−0.02), yet this decreased to 0.26 (+/−0.04) when decreasing the number of trees from 100 to 25. Similarly, an increase in number of trees to 400, decreased the performance followed by an increase when more trees were developed (Fig. 4). In contrast, average computation time per repetition did change along an increase in the number of trees, with a stronger increase for *L*. *minor* (up to 3000 seconds per repetition) and a gentler increase for *N*. *lutea* (up to 900 seconds per repetition) (Fig. 4). A clear trade-off between performance and computation time was absent, yet for each macrophyte a specific number of individual trees could be selected. For *L*. *minor* and *N*. *lutea* a relatively higher and more stable performance was observed for 100 individual trees (Fig. 4). For *S*. *polyrhiza*, the highest performance was observed for 25 individual trees (kappa = 0.22 +/−0.03), along with a computation time of 49 seconds per repetition. A slightly lower and more stable performance for *ntree* = 100 (180 seconds per repetition) was considered to be a valid compromise between performance, stability and computation time.

Since only three variables remained, increasing the default value for *mtry* (number of variables to be considered for each split, equal to two for three variables) up to 3 lead to the development of individual classification trees and increased the correlation among the models. By decreasing the default value from 2 to 1, the algorithm was basically told which variable to consider, as there were no other options. Hence, for further analyses, the default value was maintained.

### Prediction error

**Figure 5:**
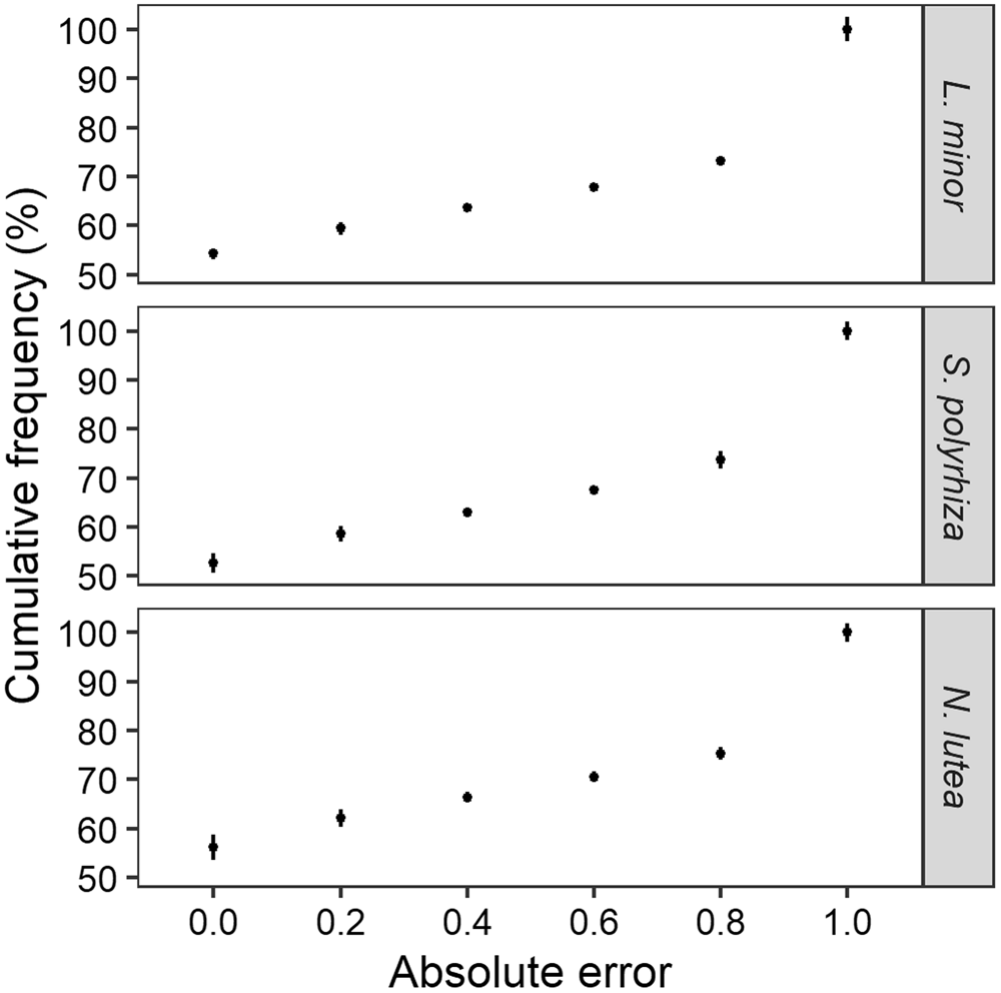
Average absolute error between observations and predictions for the evaluation data set. At least 50% of the cases are predicted correctly (error equal to zero), while about 30% are predicted incorrectly (error equal to one), with the remainder in between both values. Errors were calculated based on predictions of the evaluation data set, via random forests trained on data with 18% missing values, no outlier or correlated variable removal and followed by imputation of the median value. A 5-fold cross-validation approach was applied and repeated 10 times, with number of individual trees set to 100.

In general, random forest trained with the M18 data, without outlier or correlated variable removal and followed by imputation of the median value provided a correct prediction (absolute error equal to zero) when applied to the evaluation set in at least 50% of the cases (settings: *ntree* = 100, 5-fold CV, 10 repetitions). On the other hand, 30% of the cases were predicted completely incorrectly (absolute error equal to one), with the remainder of the cases equally distributed between both extremes. This is represented in Fig. 5, showing a somehow linear increase in cumulative frequency between an error value of 0.0 and an error value of 0.8, followed by a sudden increase of about 30% up to 1.0. A similar pattern was observed for each of the three macrophytes

### Variable influence

**Figure 6:**
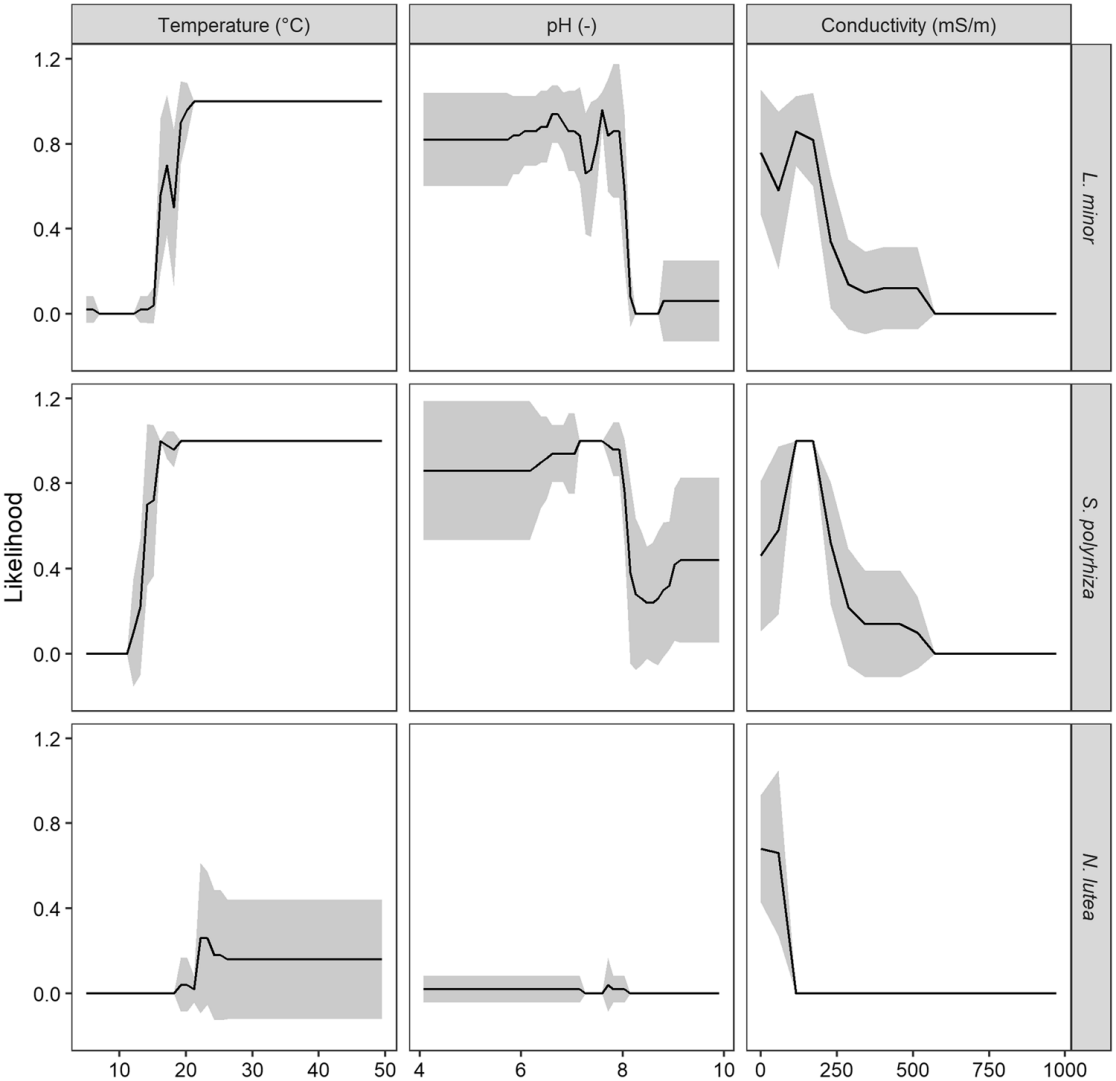
Influence of remaining variables on likelihood of macrophyte presence. Likelihood values were calculated as predictions in which one variable’s value gradually increased, while the remaining variables’ values were fixed at their median value. Random forests were trained with data of which 18% was missing, without outlier or correlated variable removal and followed by median imputation, while applying 5-fold cross-validation. In total, 10 repetitions were performed and likelihood values were averaged (black lines), with grey zones representing the standard deviation over these 10 repetitions. The number of individual trees was equal to 100 for each macrophyte.

Temperature, pH and conductivity were the three remaining variables within M18. While ranging one variable from its lowest observed value up to its highest observed value, with the other two variables maintaining their mean values, the general influence of each variable was inferred. As no outliers were removed from the initial data set, wide ranges for each of the variables were observed and included in the likelihood calculations. To improve visualisation, narrower ranges were therefore identified and depicted in Fig. 6, while the complete ranges can be seen in Fig. S4. Increasing temperature had a positive effect on the likelihood of all three macrophytes (especially when higher than 20 °C), yet still resulted in average likelihood values below 0.5 for *N*. *lutea* (settings: *ntree* = 100, 5-fold CV, 10 repetitions) (see Fig. 6). In contrast, elevated conductivity values decreased the likelihood of each macrophyte, with an optimal value around 50 mS m^−1^. Lastly, pH seemed to positively affect likelihood when values were intermediate, i.e. between 6.9 and 8, and showed a clear drop in likelihood for *L*. *minor* and *S*. *polyrhiza* when values become higher than 8. No clear effect on *N*. *lutea* could be inferred. Grey zones represent the variability of the likelihood values within the ten repetitions and showed that special care should be taken when considering high pH values for *S*. *polyrhiza*, with a value of 0.4+/−0.35, thereby crossing the frequently-applied threshold of 0.5 to differentiate between presence and absence. In contrast, extreme variable values either clearly support (e.g. high temperatures for both *L*. *minor* and *S*. *polyrhiza*) or counteract (e.g. high conductivity values for each macrophyte) the presence of the considered macrophytes as no standard deviation in likelihood is observed (Fig. 6).

## Discussion

### Model development

Extensive data sets represent a unique source of valuable information, yet often contain a high amount of missing data. Via data reduction, the relative amount of missing data can be reduced at the expense of variables, observations or a combination of both. With respect to the Limnodata, an intermediate reduction to 40% missing data did not provide sufficient performance, regardless of the applied imputation method, and required a further decrease down to 18%. Simultaneously, this lead to a decrease in number of variables down to three, limiting further reduction at the expense of observations. With this data, fair model performances (0.21–0.40^33^) were obtained in general, regardless of the applied data preprocessing and imputation method. Yet, the potential presence of pseudo-absences might have resulted in optimistic true negative or pessimistic false positive values, thereby affecting reported model performance. Moreover, this bias protrudes to the level of predictions by inflating the degree of false negatives (i.e. species predicted to be absent, while being present). The effect of unobserved presences being reported as absences on random forest performance constitutes an interesting topic for further research, but was considered to be out of the scope of this article.

Outlier and correlated variable removal is a frequently applied step within the framework of developing data-driven habitat suitability models, thereby influencing the available data for pattern extraction. With a decreasing number of observations, the influence of a single outlier increases, especially when missing data are replaced by the mean value. Presence of outliers also affects the final model, yet due to the methodology of selecting a subset of variables for each split, random forests are less prone to be affected by outlier presence. This suggests that random forests do not require the removal of outliers, but needs further testing to be confirmed.

Related to the imputation methods, two approaches have been frequently applied in the past and are sporadically mentioned in more recent literature: average and median value. Both methods rely on univariate statistics, of which the selection depends on the distribution of the considered variable^34^. Contrasting these univariate approaches, *k*-nearest neighbours (kNN) and *missForest* (mF) represent multivariate techniques, relying on the intrinsic associations among the available data. Both kNN and mF have been reported to clearly outperform univariate imputation methods like mean and median imputation^31,35^, however fail to support higher model performances in this specific case. This suggests that the proportion of missing data might still have been too high to unravel the intrinsic patterns and interactions. As a result, the imputed data is highly likely to be close to a central value (e.g. mean, median) of the considered variable, hence resulting in similar performances. Solving this issue requires more observations to be removed, which, under the ‘missing completely at random’ assumption, intrinsically leads to a decrease in prevalence. With the negative effect of decreased prevalence on random forest performance, one ends up with the dilemma of either removing observations or maintaining model accuracy. For instance, reducing the proportion of missing data by removing observations, positively affects model performance and interpretation (less missing data)^35,36^, but potentially decreases model accuracy on the other hand (less observations)^19,30,35,37–39^. So far, the relative importance of both processes are considered to be case-specific, without a single guideline on how number of observations, number of variables and proportion of missing data can be appropriately combined to provide optimal model performances. With limited discrepancy among the applied imputation techniques it is deemed important to have a closer look at the efficacy of each imputation technique for different combinations of missing data and minimum number of variables by considering a complete set of data and artificially removing data points.

Increasing the number of individual trees resulted in similar performances and an increase in computation time, thereby contrasting literature. For instance, Vezza, *et al*.^29^ applied a range of *ntree* values and found that error stabilisation occurred between 1500 and 2500 trees. Similarly, Kubosova, *et al*.^28^ applied random forests for determining the steering environmental variables for macroinvertebrates and determined that out of the *ntree* range, 500 trees resulted in a stable performance. Still, both did not consider the computation time, although it allows for a practical trade-off between performance, number of repetitions and required time for model development. Additionally, the number of variables to be considered for each split (*mtry*) plays a role in the overall required time, as a higher number increases both the complexity and the potential of including an informative variable^40^. When selecting all variables to be considered, the random forest basically becomes an ensemble of ‘bagged’ classification trees, thereby decreasing computation time (as variables do not have to be selected randomly), but simultaneously increasing the correlation among the individual trees^22,40^. At the other extreme, selecting only one variable does not allow for the model to select the most informative variable as it has already been randomly selected. The optimal settings within this case (*ntree* = 100) suggest that the default settings mostly increase computation time, with case-specific effects on performance values when considering altered settings. Therefore, testing a range of model settings when developing a habitat suitability model supports a more stable result, by optimising performance and computation effort when high numbers of repetitions or *k*-fold cross-validation are preferred. Hence, a preliminary analysis provides a useful tool for parameter value selection, as an improvement in model performance does not always follow an increase in forest size or number of variables (and related computational cost)^28,41^.

### Ecological relevance

Only three variables remained in the selected data set, as all others were removed for decreasing the overall amount of missing data. Observations for temperature, pH and conductivity supported the development of fair random forests, while supporting our expectations only partly. For instance, Svitok, *et al*.^42^ identified pH, conductivity, turbidity, and substrate composition as the most influential variables towards macrophyte diversity, while Ciecierska and Kolada^10^ introduced the multimetric Ecological State Macrophyte Index (ESMI) and observed that the index was highly correlated with water transparency (representing light availability) and nutrient concentrations. Additionally, Bornette and Puijalon^2^ identified temperature, CO_2_, nitrogen, phosphorus, substrate composition, water movements and potential disturbances as important variables, though stressed that preferred abiotic conditions vary among different macrophytes. This illustrates the difficulty of defining a single physical-chemical variable that influences macrophyte presence in the context of macrophyte-specific response behaviour and a wide set of influential variables^37,42^, highlighting the need for an overarching, holistic management approach that aims at restoring both the physical-chemical and the hydromorphological conditions^43^. The primary focus should remain the reduction of nutrient concentrations, as this will limit the growth of algae and related turbidity increase (hence, combatting high nitrogen levels and limited transparency). As such, existing restoration plans aiming at obtaining a clear water state, which in a later stage will be maintained by the macrophytes themselves^8^ should not be abandoned but expanded to a wider area. The subsequent optimisation (i.e. fine-tuning of the environmental conditions) of all physical-chemical variables allows one variable to fluctuate without drastically changing the presence of macrophytes.

In conclusion, our study showed that the proportion of missing data has a clear effect on subsequent model development. Removal of both observations and variables to decrease the relative amount of missing data reduced the overall number of data points drastically, especially due to a high degree of variable removal. Best performing models were obtained when the proportion of missing data was relatively limited, no data preprocessing was performed and a univariate imputation technique was applied, thereby highlighting the importance of assessing the data structure of each case study in detail. High temperatures, low conductivity values and intermediate pH values showed to positively influence the likelihood of *Lemna minor*, *Spirodela polyrhiza* and *Nuphar lutea*, while highlighting the necessity of holistic water management to restore natural conditions.

## Methods

### Data preprocessing

An extensive dataset was obtained from the Dutch Foundation of Applied Water Research (STOWA), i.e. the Limnodata Neerlandica^44^ and contained hydromorphological, physical-chemical and biological information on both a spatial and temporal scale. Data was collected from 1980 onwards, throughout The Netherlands, with the majority of sampled water body types being lotic waters, lakes, canals and ditches^45^. As the Limnodata Neerlandica was created as a combination of different data sets, a variety of techniques were applied to collect macrophyte-related information, including the Tansley-scale, the Braun-Blanquet method and the basic indication of presence^45^. This information was hence transformed into a presence/absence statement. Sampling location and time were combined to determine unique samples containing information on both the physical-chemical situation and macrophyte presence. Despite being very extensive, a high degree of missing data was obtained, requiring data reduction to increase the degree of information within the data set. Variables that contained less than a predefined number of observations, were removed from the data set, thereby reducing the overall number of missing data. As this influences the final data set, three threshold values were selected a posteriori out of a range of values (starting at 10% of the total number of observations), depending on the degree of missing data reduction while aiming to preserve as many observations as possible. Secondly, each dataset was subjected to either (i) no preprocessing, (ii) outlier removal, (iii) removal of correlated variables or (iv) outlier removal followed by removal of correlated variables, resulting in a total of 12 data sets. Values were assumed to be outliers when belonging to the 5% most extreme data of the considered variable. Observations with one or more outliers were subsequently removed. Correlation-based variable removal occurred when high correlation values (>0.7 or <−0.7) were obtained, resulting in the removal of the variable with the highest number of missing data.

### Data imputation and macrophyte selection

Missing values were replaced by either (i) the median value, (ii) the average value, or via (iii) *k* nearest neighbours (kNN) or (iv) the *missForest* algorithm^30–32^, providing a total of 48 data sets. Imputation of the median or average value represents a simple, univariate imputation method, being frequently applied in the past, yet criticised for ignoring the associations between variables^32,35^. These associations are taken into account when applying a multivariate approach like *k* nearest neighbours (kNN) or *missForest*.

The kNN technique relies on the calculation of inter-observation distances (e.g. calculated as Gower similarities), followed by the identification of observations resembling the observation with missing data the most (its so-called neighbours). Subsequently, a mean or median value of the surrounding *k* neighbours is imputed (optionally after weighing according to their distances) to replace the missing value^32^. Alternatively, a user-defined function can be specified instead of the default univariate approaches. Specific settings for kNN-based imputation entailed the calculation of the distance-weighted median value, taking into account the five nearest neighbours. Alternative settings were not considered as these merit a different discussion.

Lastly, the *missForest* algorithm relies on the identification of patterns within the data via the development of random forests^31^. For each variable, a random forest (see further) is created and iteratively updated until the out-of-bag error starts to increase again (or the user-defined maximum number of iterations is reached). Specific settings for *missForest* entailed a maximum of 10 iterations, each consisting of 100 trees to be developed with data being randomly sampled without replacement. Similar to kNN, no alternative settings were considered.

Each data set was subsequently linked with the observed presences of three macrophytes, with macrophyte absence being assumed when presence was not reported. Macrophyte selection was based on their prevalence within the original data, as to determine the associated effect of data prevalence. Presence/absence data was preferred over abundance or percentage data as the latter is more prone to errors, despite containing more information and potentially providing a higher degree of performance^46–48^. Nevertheless, the authors would like to point out that, by assuming absence when presence is not reported, potential bias is introduced into the data as pseudo-absences. This affects both the reported prevalences and the variable-specific effects on the likelihood of macrophyte presence, hence requiring careful interpretation of the obtained results.

### Model characteristics and development

During the random forests model development, a series of individual decision trees is created, resulting in a response that averages all individual trees. Each individual tree is trained with a subset of the initial training dataset and, prior to each split, a subset of the considered variables is used (default $mtry=\sqrt{V}$, with *V* the number of variables). Subsequently, the Gini node impurity $(I(p)=\sum _{k}{p}_{k}\cdot (1-{p}_{k})$, with *k* the number of classes and *p*_*k*_ the fraction of instances classified within class *k*) is calculated to determine the most informative split (i.e. lowest Gini node impurity)^26^. For each split, a new combination of variables is considered, for which the optimal splitting value is sought for within the random subspace. Finally, the resulting individual tree is assessed for its performance with data that was not used for model training (the out-of-bag data). This process of single tree development is repeated multiple times to end up with a series of models consisting of a predefined number of trees (default *ntree* = 500). Because of the random selection of variables for each split, the developed classifiers are only limitedly correlated, allowing to combine (i.e. bagging) the individual responses into an average response^26,49^. Hence, the final response of the model is determined based on a majority vote of all individual trees, with ties assigned randomly^22,50^. Advantages of the random forest technique include limited overfitting, robustness towards noise, no need for an a priori assumed variable distribution and the possibility to determine variable importance^20,29,50^. Yet, as the latter is reported to be flawed when variables have different scales or number of categories^49^, conditional random forests were applied to all cases, allowing to identify steering variables when the data contained more than five variables.

### Model settings and development

The final model, its performance, the variables used and the required computation time are influenced by setting the model parameters. For instance, large random forests (i.e. high number of individual trees) might only increase the computational costs without improving the performance^41^. Similarly, the number of variables to be considered for each split (*mtry*) affects the ease of finding a proper split value. Therefore, a range of forest sizes was defined subjectively, starting at 25 up to 1000 individual trees, totalling six different settings. In contrast, the range and values of the *mtry* parameter depended on the number of remaining variables within the data set and was not defined in advance. By decreasing the number of variables to be considered for each split (*mtry*), the environmental space to look into for extracting patterns is reduced, hence increasing the risk of eliminating a potentially important variable (or interaction), yet simultaneously decreasing data complexity^40^. Changes in parameter values were applied after determining which data set provided the best performance when applying default parameter values (except for *ntree*, which was set to be equal to 100 instead of 500). Subsequently, an optimal macrophyte-specific value for *ntree* was determined, followed by *mtry*. When developing models along the parameter ranges, the average computation time for a single repetition was registered, allowing a trade-off evaluation of performance versus computation time. Calculations were performed with an Intel® Core™ i3-4030U, 1.9 GHz CPU and 8 GB RAM.

### Performance and variable importance

Models were assessed on their performance when dealing with an external data set (20% of the data that was not used for model development), which was randomly subsampled from the imputed data. Due to the random subsampling of the original data, the evaluation set was assumed to represent a similar prevalence as the original data. Moreover, an internal validation was performed by applying a 5-fold cross-validation with each fold being sampled randomly without replacement and being characterised by a 50% prevalence. Selection of 5-fold cross-validation was based on a preliminary analysis over three *k*-values (3, 5 and 10) for the biggest data set, without data preprocessing and with univariate imputation of the median for *L*. *minor*, repeated 10 times with *ntree* = 100 (see Fig. S5). As each dataset to be used in model training or validation was defined to represent a 50% prevalence^25,39,51^, a higher reduction in data was needed for species with a lower prevalence.

Three different metrics were chosen for assessing model performance: Cohen’s kappa (κ)^52^, sensitivity (Sn), and specificity (Sp), based on the confusion matrix (see Table S2 and Equations S2, S3 and S4). Cohen’s kappa was chosen over accuracy (correctly classified instances, CCI, Equation S1), as the evaluation set contained a similar prevalence distribution as the original data (i.e. lower than 50%), hence influencing the final CCI score^53^.

Model development occurred according to the following process: (1) define the model settings to be applied, (2) select a preprocessed and imputed data set, randomly subsample 20% and store this as an evaluation set, (3) out of the remaining 80%, create 5 equal-sized data sets (sampled randomly and without replacement), taking care that each individual data set represents a 50% prevalence of the considered macrophyte, (4) perform 5-fold cross-validation, while determining the results of validation and evaluation for each fold separately, (5) calculate the average validation performance, evaluation response and evaluation performance, (6) repeat this procedure (starting from (2)) in total 10 times to include the effect of data availability. Conditional random forests were developed in RStudio^54,55^ with the additional *partykit* package^56,57^ (which relies on the *randomForest* package). Data imputation was performed with the *Hmisc*, *VIM* and *missForest* package^58–60^. The range of obtained datasets and selected settings was used to train and evaluate random forests as recommended by Everaert, *et al*.^25^, Goethals, *et al*.^61^ and Araújo and Guisan^38^.

Additionally, the absolute differences between the observed and predicted probabilities of occurrence were determined in order to investigate the prediction error. Errors were determined based on the individual response of each fold and subsequently averaged, resulting in a range of six discrete error values (0.0, 0.2, 0.4, 0.6, 0.8 and 1.0). A high cumulative frequency for *error* = 0.0 describes a model able to predict specific situations accurately (distance close to 0), while a high difference between 0.8 and 1.0 represents a high degree of completely incorrectly classified cases.

Based on the aforementioned analyses, an approach to determine variable influence on macrophyte presence was inferred based on the effect of (i) data reduction, (ii) data preprocessing, (iii) imputation technique and (iv) model settings on model performance. At each level, the approach resulting in the highest performance was selected and used for training a new random forest. With this model, predictions were made of the macrophytes’ likelihood of presence along the observed range of a single variable, while other variables maintained their observed median value. Model development entailed a five-fold cross-validation, with predictions being performed ten times. Predictions of a single random forest were reported as a binary presence/absence response, and were averaged over all repetitions.

## Electronic supplementary material

Supplementary Information

## Data Availability

The dataset (Limnodata Neerlandica) analysed during the current study is available on the STOWA website^44^, https://www.gbif.org/dataset/37f48e00-1fe8-11dc-b461-b8a03c50a862.

## References

[1] Butcher RW (1933). Studies on the Ecology of Rivers: I. On the Distribution of Macrophytic Vegetation in the Rivers of Britain. J. Ecol..

[2] Bornette G, Puijalon S (2011). Response of aquatic plants to abiotic factors: a review. Aquat. Sci..

[3] Dennison WC (1993). Assessing Water Quality with Submersed Aquatic Vegetation. Bioscience.

[4] Choi J-Y (2014). Role of macrophytes as microhabitats for zooplankton community in lentic freshwater ecosystems of South Korea. Ecological Informatics.

[5] Marion L, Paillisson J-M (2003). A mass balance assessment of the contribution of floating-leaved macrophytes in nutrient stocks in an eutrophic macrophyte-dominated lake. Aquat. Bot..

[6] MEA. Ecosystems and Human Well-Being: Synthesis. (World Resources Institution, Washington, D.C., 2005).

[7] Engelhardt KAM, Ritchie ME (2001). Effects of macrophyte species richness on wetland ecosystem functioning and services. Nature.

[8] Bakker ES, Sarneel JM, Gulati RD, Liu Z, van Donk E (2013). Restoring macrophyte diversity in shallow temperate lakes: biotic versus abiotic constraints. Hydrobiologia.

[9] Hilt S (2006). Restoration of submerged vegetation in shallow eutrophic lakes – A guideline and state of the art in Germany. Limnologica - Ecology and Management of Inland Waters.

[10] Ciecierska H, Kolada A (2014). ESMI: a macrophyte index for assessing the ecological status of lakes. Environ. Monit. Assess..

[11] Hatten J, Batt T, Connolly P, Maule A (2014). Modeling effects of climate change on Yakima River salmonid habitats. Clim. Change.

[12] Domisch S (2013). Modelling distribution in European stream macroinvertebrates under future climates. Global Change Biol..

[13] Kemp WM (2004). Habitat requirements for submerged aquatic vegetation in Chesapeake Bay: Water quality, light regime, and physical-chemical factors. Estuaries.

[14] Mount NJ (2016). Data-driven modelling approaches for socio-hydrology: opportunities and challenges within the Panta Rhei Science Plan. Hydrological Sciences Journal.

[15] Lawson CR, Hodgson JA, Wilson RJ, Richards SA (2014). Prevalence, thresholds and the performance of presence–absence models. Methods in Ecology and Evolution.

[16] Kampichler C, Wieland R, Calmé S, Weissenberger H, Arriaga-Weiss S (2010). Classification in conservation biology: A comparison of five machine-learning methods. Ecological Informatics.

[17] Van Echelpoel, W. *et al*. In *Developments in Environmental Modelling* Vol. Volume 27 (eds Sovan Lek Christophe Baehr Young-Seuk Park & Jørgensen Sven Erik) 115-134 (Elsevier, 2015).

[18] Gobeyn S, Volk M, Dominguez-Granda L, Goethals PLM (2017). Input variable selection with a simple genetic algorithm for conceptual species distribution models: A case study of river pollution in Ecuador. Environ. Model. Software.

[19] Stohlgren TJ (2010). Ensemble Habitat Mapping of Invasive Plant Species. Risk Anal..

[20] Elith J, Graham CH (2009). Do they? How do they? WHY do they differ? On finding reasons for differing performances of species distribution models. Ecography.

[21] Marmion M, Parviainen M, Luoto M, Heikkinen RK, Thuiller W (2009). Evaluation of consensus methods in predictive species distribution modelling. Divers. Distrib..

[22] Cutler DR (2007). Random Forests for Classification in Ecology. Ecology.

[23] Boets P, Lock K, Goethals PLM (2013). Modelling habitat preference, abundance and species richness of alien macrocrustaceans in surface waters in Flanders (Belgium) using decision trees. Ecological Informatics.

[24] Hoang TH, Lock K, Mouton A, Goethals PLM (2010). Application of classification trees and support vector machines to model the presence of macroinvertebrates in rivers in Vietnam. Ecological Informatics.

[25] Everaert, G., Pauwels, I., Bennetsen, E. & Goethals, P. L. M. Development and selection of decision trees for water management: Impact of data preprocessing, algorithms and settings. *AI Commun*., 1–13 (2016).

[26] Archer KJ, Kimes RV (2008). Empirical characterization of random forest variable importance measures. Comput. Stat. Data Anal..

[27] Rokach, L. *Data mining with decision trees: theory and applications*. Vol. 69 (World scientific, 2008).

[28] Kubosova K, Brabec K, Jarkovsky J, Syrovatka V (2010). Selection of indicative taxa for river habitats: a case study on benthic macroinvertebrates using indicator species analysis and the random forest methods. Hydrobiologia.

[29] Vezza P, Muñoz-Mas R, Martinez-Capel F, Mouton A (2015). Random forests to evaluate biotic interactions in fish distribution models. Environ. Model. Software.

[30] Oba S (2003). A Bayesian missing value estimation method for gene expression profile data. Bioinformatics.

[31] Stekhoven DJ, Bühlmann P (2012). MissForest—non-parametric missing value imputation for mixed-type data. Bioinformatics.

[32] Troyanskaya O (2001). Missing value estimation methods for DNA microarrays. Bioinformatics.

[33] Landis JR, Koch GG (1977). The Measurement of Observer Agreement for Categorical Data. Biometrics.

[34] Marvin LB, John FK (2003). Data mining and the impact of missing data. Industrial Management & Data Systems.

[35] Moorthy K, Saberi Mohamad M, Deris S (2014). A Review on Missing Value Imputation Algorithms for Microarray Gene Expression Data. Current Bioinformatics.

[36] Liew AW-C, Law N-F, Yan H (2011). Missing value imputation for gene expression data: computational techniques to recover missing data from available information. Briefings in Bioinformatics.

[37] Pulido, C., Riera, J. L., Ballesteros, E., Chappuis, E. & Gacia, E. Predicting aquatic macrophyte occurrence in soft-water oligotrophic lakes (Pyrenees mountain range). *J*. *Limnol*. **74** (2014).

[38] Araújo MB, Guisan A (2006). Five (or so) challenges for species distribution modelling. J. Biogeogr..

[39] McPherson JM, Jetz W, Rogers DJ (2004). The effects of species’ range sizes on the accuracy of distribution models: ecological phenomenon or statistical artefact?. J. Appl. Ecol..

[40] Strobl C, Malley J, Tutz G (2009). An Introduction to Recursive Partitioning: Rationale, Application and Characteristics of Classification and Regression Trees, Bagging and Random Forests. Psychological methods.

[41] Oshiro Thais Mayumi, Perez Pedro Santoro, Baranauskas José Augusto (2012). How Many Trees in a Random Forest?. Machine Learning and Data Mining in Pattern Recognition.

[42] Svitok M, Hrivnák R, Kochjarová J, Oťaheľová H, Paľove-Balang P (2016). Environmental thresholds and predictors of macrophyte species richness in aquatic habitats in central Europe. Folia Geobotanica.

[43] Haase P, Hering D, Jähnig SC, Lorenz AW, Sundermann A (2013). The impact of hydromorphological restoration on river ecological status: a comparison of fish, benthic invertebrates, and macrophytes. Hydrobiologia.

[44] Knoben, R. & van der Wal, B. In *OccurrenceDataset* (ed Dutch Foundation for AppliedWater Research) (2015).

[45] STOWA. Limnodata Neerlandica - De aquatisch-ecologische databank voor Nederland. Report No. 2001–32, 26 (2001).

[46] Howard C, Stephens PA, Pearce-Higgins JW, Gregory RD, Willis SG (2014). Improving species distribution models: the value of data on abundance. Methods in Ecology and Evolution.

[47] Gobeyn S, Bennetsen E, Van Echelpoel W, Everaert G, Goethals PLM (2016). Impact of abundance data errors on the uncertainty of an ecological water quality assessment index. Ecol. Indicators.

[48] Babyak M. A. (2004). What You See May Not Be What You Get: A Brief, Nontechnical Introduction to Overfitting in Regression-Type Models. Psychosomatic Medicine.

[49] Strobl C, Boulesteix A-L, Zeileis A, Hothorn T (2007). Bias in random forest variable importance measures: Illustrations, sources and a solution. BMC Bioinformatics.

[50] Breiman L (2001). Random Forests. Machine Learning.

[51] Liu C, Berry PM, Dawson TP, Pearson RG (2005). Selecting thresholds of occurrence in the prediction of species distributions. Ecography.

[52] Cohen J (1960). A Coefficient of Agreement for Nominal Scales. Educational and Psychological Measurement.

[53] Fielding AH, Bell JF (1997). A review of methods for the assessment of prediction errors in conservation presence/absence models. Environ. Conserv..

[54] R: A language and environment for statistical computing v. 3.3.1 (Vienna, Austria, 2016).

[55] RStudio: Integrated Development for R v. 0.99.903 (RStudio, Inc., Boston, MA, 2015).

[56] Hothorn T, Hornik K, Zeileis A (2006). Unbiased Recursive Partitioning: A Conditional Inference Framework. Journal of Computational and Graphical Statistics.

[57] partykit: A Modular Toolkit for Recursive Partytioning (2015).

[58] Hmisc: Harrel Miscellaneous v. 4.1-1 (2018).

[59] Kowarik A, Templ M (2016). Imputation with the R Package VIM. Journal of Statistical Software.

[60] MissForest: Nonparametric Missing value Imputation using Random Forest. v. 1.4 (2013).

[61] Goethals PLM, Dedecker AP, Gabriels W, Lek S, De Pauw N (2007). Applications of artificial neural networks predicting macroinvertebrates in freshwaters. Aquat. Ecol..

