# Supplementary material for: Variable importance for sustaining macrophyte presence via random forests: data imputation and model settings
Source: Sci Rep. 2018 Sep 28;8:14557. doi: 10.1038/s41598-018-32966-2 (PMC6162213; doi:10.1038/s41598-018-32966-2)
Supplement: Supplementary file 1 — Supplementary Information [file 41598_2018_32966_MOESM1_ESM.pdf]

Supplementary Information

for

Variable importance for sustaining macrophyte presence via random forests: data imputation and model settings

Wout Van Echelpoel[a,*], Peter L. M. Goethals[a]

[a] Department of Animal Sciences and Aquatic Ecology, Ghent university, Coupure Links 653, B-9000 Ghent, Belgium

* Corresponding author: wout.vanechelpoel@ugent.be

Table S1: Variables included in de random forest development. *Reported values represent the range (minimum-maximum), the distribution (First quartile-Median-Third quartile) and the Mean value.*

	Variable	Minimum	First quartile	Median	Mean	Third quartile	Maximum	#NAs (%)
60 % (N = 4327)								
	Temperature	0.00	14.50	17.30	17.19	20.00	100.00	988 (22.8)
	pH	0.0	7.3	7.7	7.7	8.1	10.9	1490 (34.4)
	Conductivity	0.0	46.0	61.0	139.8	91.8	5660.0	1658 (38.3)
	Transparency	0.00	0.25	0.40	0.53	0.60	80.00	2220 (51.3)
	Depth	0.00	0.25	0.40	0.84	0.80	20.00	3263 (75.4)
	Velocity	0.0	8.0	20.0	24.2	33.0	150.0	3334 (77.1)
	Oxygen	0.00	5.00	7.60	8.03	9.94	120.00	2150 (49.7)
	Oxygen saturation	0.0	51.0	78.0	82.3	104.0	391.0	2088 (48.3)
	BOD_5	0.0	2.0	3.1	4.6	5.4	68.0	2964 (68.5)
	Total Phosphorus	0.01	0.09	0.19	0.46	0.49	8.80	2437 (56.3)
	Phosphate-Phosphorus	0.00	0.02	0.05	0.30	0.25	12.0	2537 (58.6)
	Total Nitrogen	0.00	1.55	2.40	3.79	4.25	64.00	3314 (76.6)
	Kjeldahl-Nitrogen	0.07	1.10	1.70	2.16	2.60	20.00	2571 (59.4)
	Ammonia-Nitrogen	0.001	0.100	0.190	0.465	0.400	17.800	2236 (51.7)
	Nitrite-Nitrogen	0.0002	0.0100	0.0200	0.0878	0.0600	60.0000	2634 (60.9)
	Nitrate-Nitrogen	0.01	0.05	0.20	1.44	1.56	32.00	2457 (56.8)
	Oxidised Nitrogen	0.01	0.05	0.20	1.62	1.33	35.00	3503 (81.0)
	Chlorophyll *a*	0.0	6.0	14.0	46.3	47.0	1720.0	3205 (74.1)
	Pheophytin	0.01	6.00	10.00	26.99	23.00	571.00	3858 (89.2)

	Potassium	0.005	4.000	6.300	9.645	11.440	180.000	3140 (72.6)
	Calcium	0.04	45.00	67.00	72.90	90.00	780.00	2799 (64.7)
	Magnesium	0.01	7.60	10.00	15.59	15.00	530.00	3134 (72.4)
	Sodium	0.03	19.00	33.00	89.61	63.00	4700	3190 (73.7)
	Chloride	5.0	37.0	64.8	212.6	135.0	11600	2148 (49.6)
	Sulphate	0.08	29.63	52.00	72.24	80.00	1200	2839 (65.6)
	Bicarbonate	1.0	120.0	170.8	193.8	240.0	1590.6	3699 (85.5)
40 % (N = 4107)								
	Temperature	0.00	14.50	17.30	17.19	20.00	100.00	768 (18.7)
	pH	0.0	7.3	7.7	7.7	8.1	10.9	1270 (30.9)
	Conductivity	0.0	46.0	61.0	139.8	91.8	5660.0	1438 (35.0)
	Transparency	0.00	0.25	0.40	0.53	0.60	80.00	2000 (48.7)
	Oxygen	0.00	5.00	7.60	8.03	9.94	120.00	1930 (47.0)
	Oxygen saturation	0.0	51.0	78.0	82.3	104.0	391.0	1868 (45.5)
	Ammonium-nitrogen	0.001	0.100	0.190	0.465	0.400	17.800	2016 (49.1)
	Chloride	5.0	37.0	64.8	212.6	135.0	11600	1928 (46.9)
18 % (N = 3604)								
	Temperature	0.00	14.50	17.30	17.19	20.00	100.00	265 (7.3)
	pH	0.0	7.3	7.7	7.7	8.1	10.9	767 (21.3)
	Conductivity	0.0	46.0	61.0	139.8	91.8	5660.0	935 (25.9)

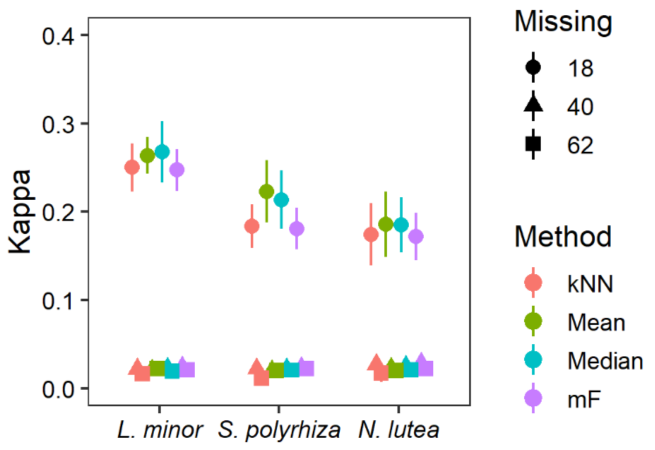

Figure S1: Effect of missing data on random forest performance, expressed as kappa values. *Higher performances are observed when the original amount of missing data is low and prevalence is relatively high. In contrast, no clear effect of imputation method on random forest performance can be observed (kNN represents* k *nearest neighbours, while mF represents the* missForest *algorithm). Depicted performances were obtained with random forest consisting of 100 trees, while running 10 repetitions and applying a 5-fold cross-validation. Selected data sets underwent outlier and correlated variable removal prior to model development.*

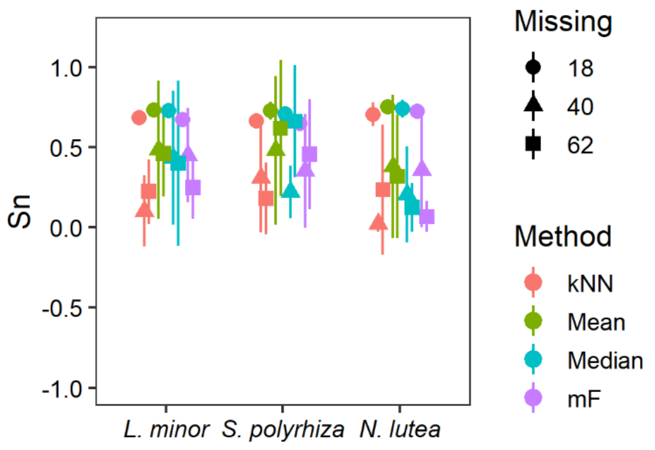

Figure S2: Effect of missing data on random forest performance, expressed as sensitivity. *Higher performances are observed when the original amount of missing data is low and prevalence is relatively high. In contrast, no clear effect of imputation method on random forest performance can be observed (kNN represents* k *nearest neighbours, while mF represents the* missForest *algorithm). Depicted performances were obtained with random forest consisting of 100 trees, while running 10 repetitions and applying a 5-fold cross-validation. Selected data sets underwent outlier and correlated variable removal prior to model development.*

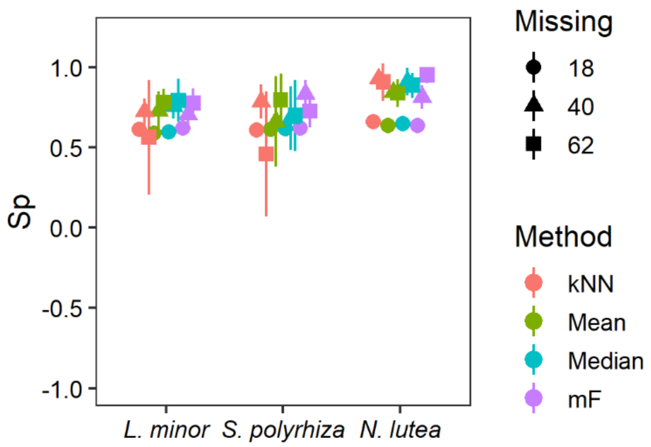

Figure S3: Effect of missing data on random forest performance, expressed as specificity. *Lower performances are observed when the original amount of missing data is low and prevalence is relatively high. In contrast, no clear effect of imputation method on random forest performance can be observed (kNN represents* k *nearest neighbours, while mF represents the* missForest *algorithm). Depicted performances were obtained with random forest consisting of 100 trees, while running 10 repetitions and applying a 5-fold cross-validation. Selected data sets underwent outlier and correlated variable removal prior to model development.*

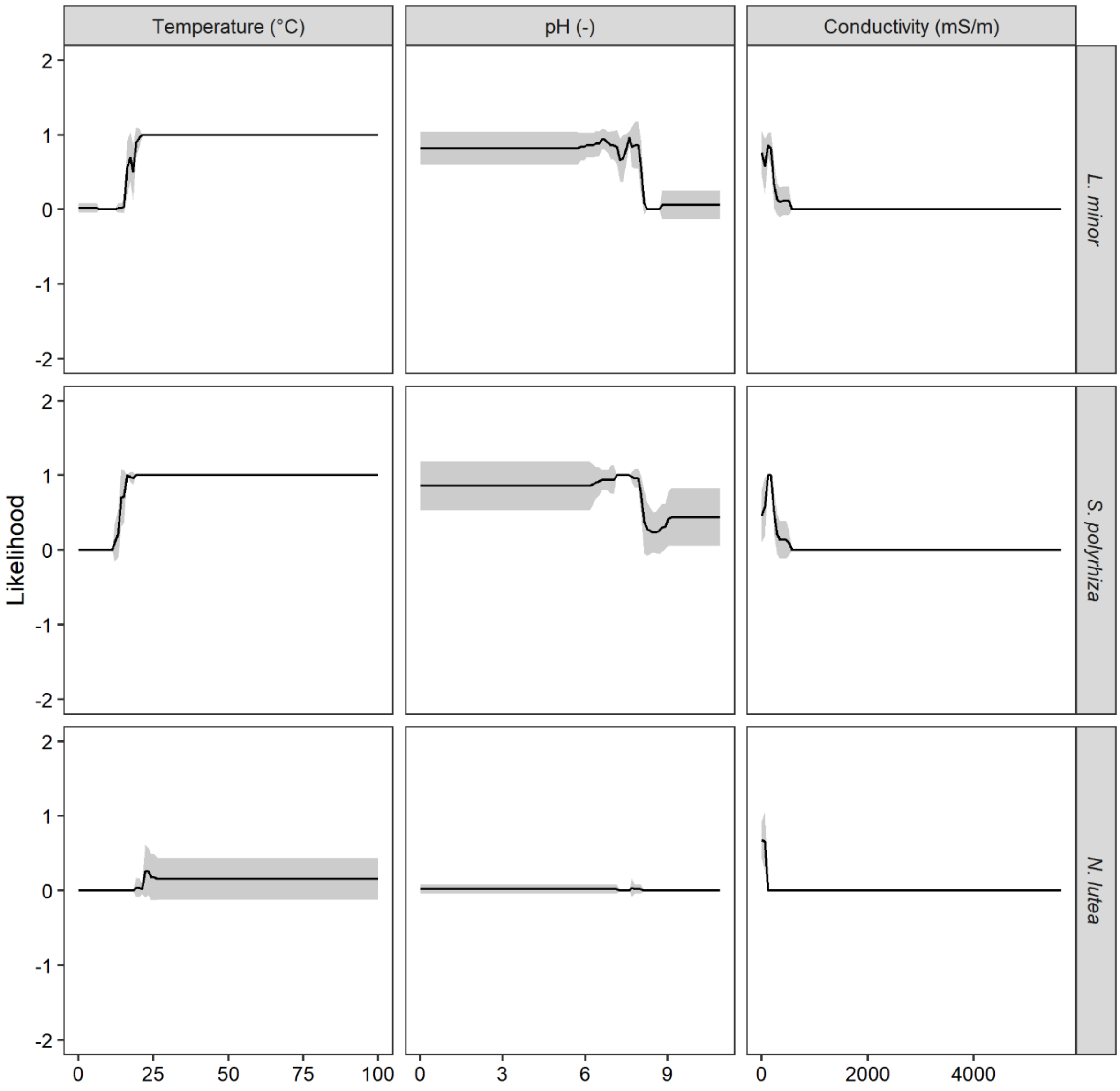

Figure S4: Influence of remaining variables on likelihood of macrophyte presence. *Likelihood values were calculated as predictions in which one variable's value gradually increased, while the remaining variables' values were fixed at their median value. Random forests were trained with data of which 18 % was missing, without outlier or correlated variable removal and followed by median imputation, while applying 5-fold cross-validation. In total, 10 repetitions were performed and likelihood values were averaged (black lines), with grey zones representing the standard deviation over these 10 repetitions. The number of individual trees was equal to 100 for each macrophyte.*

Table S2: Confusion matrix for binary observations and predictions. *TP: True Positive; FP: False Positive; FN: False Negative; TN: True Negative.*

		Observations (total = N)	
		Present	Absent
Predictions	Present	TP	FP
	Absent	FN	TN

$$CCI = \frac{TP+TN}{N} \tag{S1}$$

$$\kappa = \frac{\left((TP+TN)-\left(\frac{((TP+FN)\cdot(TP+FP)+(FP+TN)\cdot(FN+TN))}{N}\right)\right)}{N-\left(\frac{((TP+FN)\cdot(TP+FP)+(FP+TN)\cdot(FN+TN))}{N}\right)} \overset{\frac{P}{A}=1}{\Longrightarrow} 2 \cdot CCI - 1 \tag{S2}$$

$$Sn = \frac{TP}{TP+FN} \tag{S3}$$

$$Sp = \frac{TN}{TN+FP} \tag{S4}$$

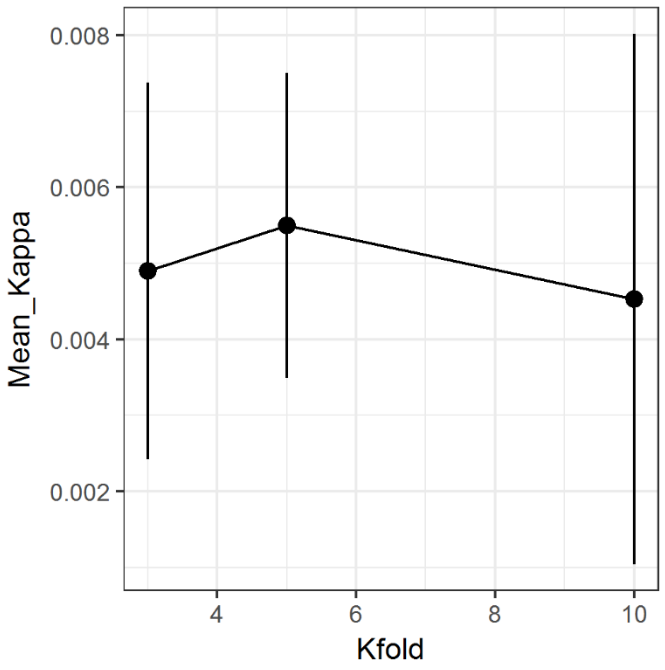

Figure S5: Effect of number of folds for **k*-fold cross-validation.*** *Performances (kappa values) were determined after training a random forest on data with 62 % missing data, no data preprocessing and imputation of the median. Random forest contained 100 individual trees and were developed for* L. minor *and repeated 10 times.*
